# Extraction, Purification and In Vitro Antioxidant Activity Evaluation of Phenolic Compounds in California Olive Pomace

**DOI:** 10.3390/foods11020174

**Published:** 2022-01-10

**Authors:** Hefei Zhao, Roberto J. Avena-Bustillos, Selina C. Wang

**Affiliations:** 1 Department of Food Science and Technology, University of California, Davis, CA 95616, USA; hefzhao@ucdavis.edu; 2 Western Regional Research Center, Healthy Processed Foods Research, Albany, CA 94710, USA; roberto.avena@usda.gov

**Keywords:** olive pomace, natural phenolic compounds, antioxidant activity, macroporous absorbing resin, hydroxytyrosol

## Abstract

Olive pomace (OP) is a valuable food byproduct that contains natural phenolic compounds with health benefits related to their antioxidant activities. Few investigations have been conducted on OP from the United States while many studies on European OP have been reported. OP of Arbequina, the most common cultivar from California, was collected and extracted by water, 70% methanol and 70% ethanol, followed by purification using macroporous absorbing resin. Results showed that the extractable total phenolic content (TPC) was 36–43 mg gallic acid equivalents (GAE)/g in pitted, drum-dried defatted olive pomace (DOP), with major contributions from hydroxytyrosol, oleuropein, rutin, verbascoside, 4-hydroxyphenyl acetic acid, hydroxytyrosol-glucoside and tyrosol-glucoside. Macroporous resin purification increased TPC by 4.6 times the ethanol crude extracts of DOP, while removing 37.33% total sugar. The antioxidant activities increased 3.7 times Trolox equivalents (TrE) by DPPH and 4.7 times TrE by ferric reducing antioxidant power (FRAP) in the resin purified extracts compared to the ethanol crude extracts. This study provided a new understanding of the extraction of the bioactive compounds from OP which could lead to practical applications as natural antioxidants, preservatives and antimicrobials in clean-label foods in the US.

## 1. Introduction

One of the most iconic and representative ingredients in the Mediterranean diet are the oils and fats of *Olea europaea* L.; the bioactive and phenolic compounds of which have been identified as the major contributions to their antioxidant and health-promoting effects by analytical and technical approaches of modern medicine as well as food science and chemistry.

Olive pomace (OP), the main residue derived from olive oil extraction, is a valuable food byproduct containing natural phenolic compounds (phenols and polyphenols) with health benefits related to their antioxidant activities. According to the Food and Agriculture Organization (FAO), the United States (mainly in California) produced 151,950 tons of olives in 2019. Based on the literature (~67.5% water and ~17.5% lipids) [1] and our preliminary experimental results of California OP that has an oil content of ~11% and total phenolic content (TPC) of 43 mg gallic acid equivalents (GAE)/g hexane defatted olive pomace (DOP), there could be as much as ~872 tons of bioactive compounds in the US OP, an equivalence of ~$79 million per year as raw material, assuming benchmark price to ~$90 per kg food-grade gallic acid. As a commercialized health food, the price of hydroxytyrosol in capsules form is currently up to $4100 per kg; therefore, the total value of the US olive pomace (fruit) extracts could be as high as about $3.6 billion per year.

Many research interests have been focused on the extraction and purification of natural phenolic compounds (NPCs) from various waste-streams of the European olive oil industry, such as olive mill wastewater (OMWW) and OP and on the bioactive abilities of the crude or purified extracts. Water among other solvents was found to be the best media for the extraction from residual oil extracted and exhausted olive pomace (EOP) in Spain, and the water-extractable TPC was 44.5 mg GAE/g EOP [2]. Aqueous extraction of OP in Portugal was successfully concentrated by reverse osmosis (RO) membranes with the highest 100% membrane retention of TPC and the lowest fouling index among two other nanofiltration (NF) membranes [3]. The TPC was increased from 0.1098 mg/mL in the feed aqueous to 1.2343 mg/mL in the concentrates, but free sugars were not removed and separated from phenols. OMWW in Italy was clarified by microfiltration (MF) and then concentrated by NF and RO, respectively [4]. While the MF slightly decreased hydroxytyrosol from 0.3733 mg/mL in feed to 0.3201 mg/mL in permeate, the hydroxytyrosol of MF permeate was increased to 1.0175 and 1.5222 mg/mL by NF and RO, respectively. In Turkey, olive leaf phenols were extracted by macroporous resins, and the Amberlite^®^ XAD7HP resin performed the highest adsorption (91%) and desorption (97%) rate of phenols based on oleuropein contents among other resins (Amberlites^®^ XAD2, XAD4 and XAD16); however, 1 g of resin was applied for the purification which was an analytical scale [5]. The purification of OP phenols coupled with the removal of sugar or other impurities has yet to be comprehensively determined and monitored. In general, the potential utilization of the bioactive compounds in OP has been far less investigated in the US than in European countries.

The objective of this study was to identify phenolic compounds, compare TPC and antioxidant activities of crude extracts from water, 70% methanol, 70% ethanol and purified extracts from pilot-scale [6] (hundreds mL of resin) and preparative chromatography columns of macroporous adsorption resin, using the most common olive cultivar in the United States. The efficacy of the removal of sugar was also monitored and reported. It is necessary to decrease sugar content in the potential OP natural ingredients because the association between sugars and the risk of obesity has been ubiquitously acknowledged by the public [7].

## 2. Materials and Methods

### 2.1. Materials and Chemicals

#### 2.1.1. Olive Material 

Fresh Arbequina olive pomace (OP) from first olive oil extraction was collected at California Olive Ranch at their facility in Artois, CA, during the 2019 harvest season and was stored at room temperature in sealed buckets for 4 h for transportation to our lab in Albany, CA, USA. Then, it was processed by the following steps reported by our research group [8]:

(1). Steam blanching. Fresh first oil extraction OP was steam-blanched for enzymatic inactivation to reduce phenolics losses. Blanching was conducted using a steam blancher at atmospheric pressure over 0.25” thick olive pomace to a final temperature of 80 °C after 3 min.
(2). Pit and skin separation. The separation of skins and pits was conducted using a 150 Langsenkamp Laboratory Separator (Warner Bodies, Elwood, IN, USA). The pomace was passed through the separator in two stages. First using a 0.060 inch hole diameter S.S. screen and then using a 0.027 inch hole diameter S.S. screen.
(3). Drum-drying. The pitted olive pomace was drum-dried on a Buflovaks Atmospheric Double Drum Dryer (Hebeler Process Solutions, Tonawanda, NY, USA), with a space of 9-10/1000” at 135 °C. Drum-drying treatments were differentiated by rotational drum speeds of 92 s/rev.
(4). Milling. To obtain smaller particle sizes, drum-dried OP samples were milled for 6 s, with a KRUPS F203 (KRUPS, Parsippany, NJ, USA) coffee mill.

#### 2.1.2. Chemicals

Amberlite^®^ XAD7HP (DuPont, Wilmington, DE, USA), chromatographic grade chemicals of hexane, water, acetic acid, sodium acetate trihydrate, methanol, ethanol, acetonitrile and analytical grade of chemicals of 2,2′-diphenyl-1-picrylhydrazyl radical (DPPH), Trolox^®^, 2,4,6-tripirydyl-Striazine (TPTZ) and ferric chloride were purchased from Fisher Scientific (Waltham, MA, USA). In addition, 96–98% (g/g) concentrated sulfuric acid, Folin–Ciocalteu reagents, sodium carbonate, phenolic compound standards of gallic acid, hydroxytyrosol, tyrosol, 4-hydroxyphenylacetic acid (4-HPA), vanillic acid, vanillin, o-coumaric acid, oleuropein, pinoresinol, cinnamic acid, caffeic acid, p-coumaric acid, ferulic acid, apigenin-7-glucoside, apigenin, luteolin-7-glucoside and luteolin were purchased from Sigma-Aldrich (St. Louis, MI, USA). Verbascoside was bought from the HWI group (Ruelzhelm, Germany). Rutin was bought from PhytoLab GmbH & Co. KG (Vestenbergsgreuth, Germany).

### 2.2. Basic Chemical Composition Analysis

#### 2.2.1. Moisture Content 

Five gram OP samples or extracts samples in triplicate were added into 5 cm diameter aluminum pans and placed in an isotherm oven (Thermo Fisher Scientific, Waltham, MA, USA) at 105 °C for 48 h till constant weight [9]. A desiccator was used for 30 min to allow dry samples to reach an ambient temperature before weighing. The final weights of each sample were recorded and used to determine the total amount of water in dry samples, considering that the amount of water is equal to the initial weight of OP samples or extracts minus the final weight of oven-dry OP samples or extracts, and then dividing by the initial weight of OP samples or extracts and multiplying by 100, its moisture content was estimated as % of the original OP samples or extracts.

#### 2.2.2. Crude Protein Content of Olive Pomace 

Protein content olive pomace was measured using an FP-628 TrueSpec N analyzer (LECO Corporation, St. Joseph, MI, USA) which referred to the method of Zhao et al. [9]. One gram of OP sample was placed into a ceramic boat for the combustion process, and each sample was analyzed by triplicate, and the nitrogen content of the OP samples was multiplied by a factor of 6.25 to convert it into protein content.

#### 2.2.3. Oil Content 

In total, 10 g of OP was determined by hexane Soxhlet extraction in a Universal Extractor (BÜCHI, New Castle, DE, USA) [9]. Each sample was performed by 30 cycles of solvent extraction and in triplicate.

#### 2.2.4. Ash Content 

In total, 3 g of each sample was placed by triplicate in crucibles and introduced into a Lindberg/Blue M box furnace (Thermo Fisher Scientific, Waltham, MA, USA) at 550 °C for 16 h, which referred to Zhao et al. [9] with some modifications. Before the final weighting, the crucibles containing the ash were carefully relocated in a desiccator to reach room temperature.

#### 2.2.5. Total Carbohydrates 

Total carbohydrates were estimated by difference.

#### 2.2.6. Total Sugar Content

The determination of total sugar content (TSC) of OP extracts referred to Zhang et al. [10] with some modifications. In brief, to 0.5 mL of 0.2 mg/L sample solution, 0.5 mL of 5% (g/g) phenol solutions was added, followed by 2.5 mL of 96% (g/g) concentrated sulfuric acid and then vortexed. The sample mixture was maintained for 10 min and then cooled in a water bath. The absorbance was measured at 490 nm by a UV-vis spectrophotometer. The sugar concentration of an unknown sample can be obtained from a calibration curve of glucose solutions prepared by a series of diluted solutions from 0 to 0.2 mg/mL, the results of total sugar content were expressed as mg glucose equivalents (GE)/g of sample. 0.2 mg/mL gallic acid standard was used as a control.

### 2.3. Extraction and Purification of Phenolic Compounds from OP

**Figure 1:**
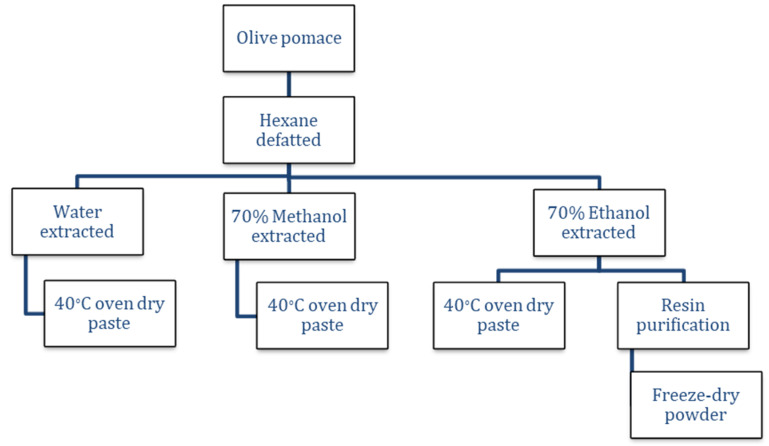
Flow diagram of the extraction and purification of pitted drum-dried olive pomace.

The flow diagram of the extraction and purification of the pitted drum-dried olive pomace powder is shown below in Figure 1.

#### 2.3.1. Extractable Phenols 

The extraction of water, 70% methanol and 70% ethanol extractable phenols of OP referred to the methods by De Bruno et al. [11] and Xu et al. [12] with some modifications. To 200 g of olive pomace (OP) in a 1000 mL beaker, 500 mL of hexane was added with gentle stirring every 30 min and stayed in a fume hood in the dark with a cover for two hours. Then, the supernatant hexane was decanted for removing oil and fat, and the hexane extractions were repeated two more times. Some of the small particles of OP inevitably outflowed from the beaker with the hexane. Then, the beaker was covered with a paper towel for overnight drying to obtain 163.8 g defatted olive pomace (DOP). Then, three of 0.25 g DOPs were extracted at room temperature (RT) by 5 mL extraction solvent of water, 70% methanol and 70% ethanol under sonication for 5 min, then stored in the dark for 3 h with shaking every 30 min. After the supernatants were poured out and collected, the precipitate was extracted again with the same procedure, which rendered the solvent to solid (from beginning) ratio as 10 mL/0.25 g, which was equal to 40:1. The extraction was centrifuged at 4 kG for 10 min at 4 °C, then the supernatant was separated and collected. All extracts were stored at RT in the dark until further analysis (normally within 5 days). Extractions were applied in triplicate for each individual OP powder-solvent combination.

#### 2.3.2. Preparation of Dry Paste of Crude Extracts of OP 

To three of 60 g DOP in a 1000 mL beaker, 300 mL of DI water, 70% methanol and 70% ethanol were added, respectively, to each of the beakers and sonicated with cover for 10 min. The three beakers were placed in a fume hood in the dark for 1 h with gentle stirring every 30 min. Then, the three upper supernatants were decanted and collected into three individual bottles and the extractions were repeated two times more to acquire a total of ~900 mL extracts for each of the three extractions media. Then, only the water extract was vacuum-filtered by the filter layer of a stomacher bag (otherwise, it was difficult to be filtrated by Whatman paper directly, due to the higher viscosity of water and more pomace swelling than those extracts in 70% ethanol and 70% methanol). The water, 70% methanol and 70% ethanol extracts were vacuum-filtered by double-layer Whatman filter papers, respectively. The three clear filtrates were collected in three 1000 mL bottles, separately and respectively, and placed in a refrigerator.

Then, the three ~900 mL extracts were concentrated by rotary evaporation at 40 °C to remove alcohol and water and decrease the volume to ~100 mL, by a Rotavapor^®^ R-300 (BÜCHI, New Castle, DE, USA). For the two extracts by 70% methanol and 70% ethanol, 100 mL water was added in each and concentrated by the rotary evaporation to ~100 mL again to thoroughly remove the alcohol out of the azeotrope; otherwise, residual alcohol was an antifreezing solution which would result in difficulty during the freeze-drying process. Low water-soluble phenols attached to the inner wall of the evaporating flask were carefully detached by a sonication bath. Then, each of the extracts was adjusted to 120 mL by DI water, and the extracts were collected into three bottles, respectively, and placed in a refrigerator. Each extract of 30 mL was poured into a 5 cm diameter aluminum pan and placed in an isotherm oven (Thermo Fisher Scientific, Waltham, MA, USA) at 40 °C for 48 h till constant weight to obtain dry pastes of OP crude extracts: 6.86 g of water extract, 6.84 g of 70% methanol extract and 7.14 g of 70% ethanol extract.

Preparation of freeze-dried powders of OP phenols extracted by 70% ethanol and purified by preparative chromatography of macroporous absorbing resin. Amberlite^®^ XAD7HP macroporous resin purification referred to the method of Jiang et al. [13]. In total, 90 mL of 70% ethanol extract after removal of ethanol was reconstituted by DI water to 600 mL and vacuum-filtered by double-layer Whatman filter papers. The clear filtrates were submitted to the XAD7HP resin column with 262.5 mL (4.8 cm diameter and 14.5 cm height) bed volumes (BV), at 1.9 BV/hour speed. The low water-soluble fractions were directedly submitted onto the top of the resin bed at the end of filtrated extracts loading absorption stage. Then, the resin bed was rinsed with 3.8 BV DI water, and the first 1/2 BV water rinsing elutes were emerged into loading absorption elutes, and those elutes were rotatory-evaporated at 40 °C and concentrated to ~100 mL and freeze-dried to obtain ~40 mL heavy syrup. The moisture content of the heavy syrup of resin elutes during absorption was tested as the previous description. To the DI water-rinsed resin bed, 3.8 BV of 70% ethanol [14] was loaded to desorb the OP phenols. Then, the desorbing elutes were rotatory-evaporated at 40 °C and concentrated to remove alcohol and decreased to ~100 mL. Then, 100 mL water was added, and the elutes were concentrated to ~100 mL again to thoroughly remove the alcohol. The concentrates were then reconstituted to 120 mL and submitted to a freeze-dryer to obtain 2.78 g resin purified extracts of OP.

### 2.4. High-Performance-Liquid-Chromatography (HPLC)-Diode-Array Detector (DAD) and HPLC-Electrospray Ionization (ESI)-Quadrupole-Time of Flight-Mass Spectrometry (Q-ToF-MS^n^) Analysis

The identification of individual phenolic compounds was implemented by an Agilent 1290 high-performance-liquid-chromatography (HPLC) system (Santa Clara, CA, USA) with an Agilent 1290 diode-array detector (DAD) which referenced Sinrod et al. [8]. An analytical C18 column (Eclipse Plus, 4.6 mm ×250 mm, 5 μm, Agilent Technologies) was used for separation. Elution was applied using mobile phase A (3% acetic acid aqueous solution) and mobile phase B (50% methanol and 50% acetonitrile). The following linear gradient was used: 0 min starting from 5% B (while 95% A, similarly hereinafter); linear increase to 30% B at 25 min; to 35% B at 35 min; to 40% B at 40 min; to 70% B at 50 min; to 100% B at 55 min, then decreasing to 5% B at 60 min and holding at 5% B for 5 min for the column equilibrium for the next injection. The flow rate was 1.0 mL/min. The injection volume was 20 µL. The DAD was set to absorbance wavelengths at 280 nm for hydroxytyrosol, tyrosol, 4-hydroxyphenylacetic acid (4-HPA), vanillic acid, vanillin, o-coumaric acid, oleuropein, pinoresinol, cinnamic acid, at 320 nm for caffeic acid, p-coumaric acid, ferulic acid, apigenin-7-glucoside, apigenin, verbascoside and at 365 nm for rutin, luteolin-7-glucoside and luteolin, respectively. Standard curves were made by each of the standard chemicals at concentrations of 10, 20, 40, 60, 80 and 100 mg/L, respectively.

Because not all the olive phenolic standards were commercially available, an Agilent 1290 HPLC coupled to an Agilent 6530 quadrupole time-of-flight mass spectrometer (Q-ToF-MS^n^) were applied to identify unknown peaks by negative mode, with instrument control and data acquisition in Agilent MassHunter Acquisition (ver. 6). The chromatographic conditions were identical to the HPLC-DAD. The collision energy (CE) was 15 eV. The unknown phenolic compounds were identified based on their MS and MS/MS fragments compared to the report of Peralbo-Molina et al. [15] and the MassBank at https://massbank.eu/MassBank.asp (accessed on 12 September 2021). Only the XAD7HP resin purified freeze-dried powders were diluted 500 times in 70% methanol and injected to HPLC- Q-ToF-MS^n^, because most of sugar and polar impurities from 0 to 14 min were eliminated by the resin from the purified extracts which facilitated the mass identification.

### 2.5. Total Phenol Content and Antioxidant Activities Analysis

#### 2.5.1. Total Phenolic Content 

The total phenol content (TPC) was determined by Folin–Ciocalteu assay which referred to the method of Zhao et al. [16]. In total, 50 μL extracted sample was added to 3 mL DI Water, 250 μL Folin–Ciocalteu reagent, 750 μL 20% sodium carbonate and 950 μL DI water. This total of 5 mL solution was incubated for 30 min in 40 °C water base, and then 200 μL solution out of 5 mL was pipetted into a microplate plate with 96 wells (Corning Incorporated, Corning, NY, USA), then determined by absorption at 760 nm in a Synergy H1 microplate reader (BioTek, Winooski, VT, USA). Here, 0.1–1 mg/mL gallic acid (GA) was utilized as a standard for the calibration curve, and the results were expressed as gallic acid equivalents (mg GAE/g DM, mg gallic acid/g of sample). Samples included DOP extracts, DOP dry pastes and freeze-dried powder.

#### 2.5.2. 2,2′-Diphenyl-1-picrylhydrazyl Radical (DPPH) Assay 

The total antioxidant activity by DPPH assay was implemented based on the slightly modified method of Zhao et al. [16]. In total, 195 μL methanolic solution of DPPH (MW 394.32 Da, 2.108 mg/100 mL methanol, Abs = 0.589) was added to 5 μL of the 0.2 mg/mL sample in 96 cuvettes microplate (Corning In., Corning, NY, USA). The absorbance of the remaining DPPH was determined at 515 nm after 30 min dark incubation at room temperature (RT). The percentage inhibition of DPPH of the test sample and known solutions of Trolox were calculated by the following formula: % Inhibition = 100 × (A0 − A)/A0, where A0 is the absorbance of DPPH methanolic solution without any inhibition, which was equal to the beginning absorbance at 515 nm, acquired by measuring the same volume of solvents of both extracted sample and the methanol solution of DPPH and A is the final absorbance of the test sample at 515 nm in a Synergy H1 microplate reader (BioTek, Winooski, VT, USA). Blank was made with 5 μL 70% methanol and 195 μL methanol without DPPH. The calibration curve between % Inhibition and known solutions of Trolox was then established. The radical scavenging activities of the test samples were expressed as Trolox equivalent (TrE) antioxidant capacity (μmol TrE/g of sample, MW of Trolox: 250.29 g/mol) on their percentage inhibitions. Trolox standard solutions were prepared at a concentration ranging from 0.1 to 1.0 μmol/mL. Samples included DOP dry pastes and freeze-dried powder.

#### 2.5.3. Ferric Reducing Antioxidant Power (FRAP) 

The FRAP assay was implemented according to the method of D’Amato et al. [17]. The fresh working FRAP solution was prepared by mixing: (1) 10 mM 2,4,6-tripirydyl-Striazine (TPTZ, MW 312.33 Da) dissolved in 40 mM HCl; (2) 20 mM ferric chloride; and (3) 300 mM acetate buffer at pH 3.6, with a ratio of 1:1:10, respectively. The FRAP was kept at 37 °C before use. In total, 50 μL of 0.2 mg/mL antioxidant solution was added to 950 μL of FRAP solution, then the mixture was incubated at 37 °C in the dark for 30 min. In addition, the absorbance was measured at 595 nm in a Synergy H1 microplate reader (BioTek, Winooski, VT, USA). Ethanol and Trolox solution (0.1–1.0 μmol/mL) were used for positive control and the standard curve, respectively. The results were expressed as μmol TrE/g of sample which included DOP dry pastes and freeze-dried powder.

### 2.6. Statistical Analysis

Triplicate data were analyzed by multiple comparison tests with Fisher’s least significant difference (LSD, *p* < 0.05) method by R software. Heatmap cluster analysis was implemented in MATLAB 2020a (The Mathworks Inc., Natick, MA, USA).

## 3. Results and Discussion

### 3.1. Chemical Composition in Olive Pomace

The basic chemical composition of pitted drum-dried olive pomace (OP) is reported in Table 1. The total carbohydrates estimated by difference was the major component which was up to 66.15%. In addition, the olive contained 11.72% residual oils. The results were in line with other works that reported total carbohydrates as the major component, including OP from Portugal containing 84.9% total carbohydrates [18], although it most likely contained pits and skins that increased their total carbohydrates. Our pitted drum-dried OP, obtained from OP pulp, also indicated that soluble saccharides could be the major impurity of the olive pomace extracts for phenolics extraction.

### 3.2. Total Phenolic Contents in Olive Pomace and Extracts

**Figure 2:**
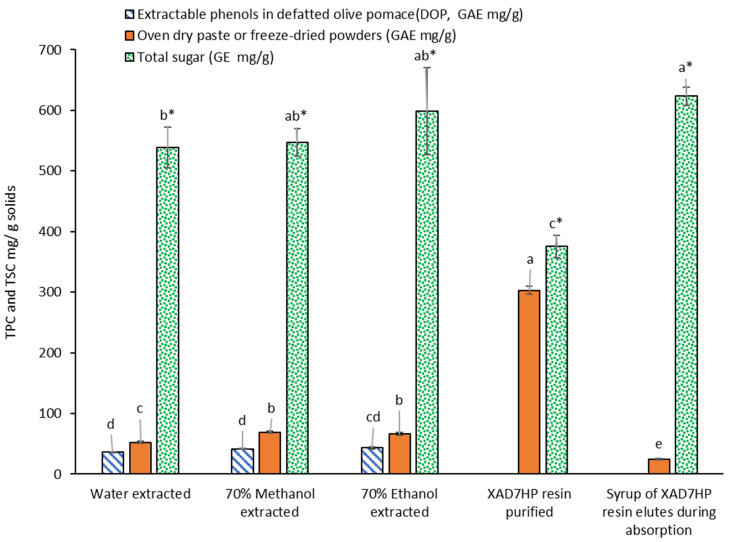
Comparison of total phenolic content (TPC) expressed by gallic acid equivalents (GAE) and total sugar content (TSC) expressed by glucose equivalents (GE) from different extraction methods and purified steps, different letters indicate significantly different, *p* < 0.05. Note: 0.2 mg/mL gallic acid standard was used as the control of the determination of total sugar content, and no interference from gallic acid was observed. Significant differences were only compared in either GAE without “*” on significant markers or GE with “*” on significant markers. Syrup of the resin elutes during sample absorption after freeze-drying still had 36.60 ± 0.17% moisture, and data were reported to dry matters (DM).

As shown in Figure 2, extractable TPC in DOP was 36.49 ± 0.29 mg GAE/g (DOP solids) by water extraction, 41.68 ± 0.95 mg/g by 70% methanol and 43.25 ± 2.08 mg/g by 70% ethanol; 70% ethanol was selected to be followed by resin purification for its highest phenolic extraction and nontoxicity food-grade potential. A similar TPC range (30–40 mg GAE/g) in exhausted olive pomace (EOP) in Spain has been reported using a variety of combinations of water-solvents extraction, and the TPC extracted by solvents was normally higher than that by water and acidified water [2]. In addition, another similar TPC range in caffeic acid equivalent (CAE) has been reported in an Italy OP, which was 38–52 mg CAE/g [19]. However, to the best of our knowledge, this is the first report on the extractable TPC range (36–43 mg GAE/g DOP solids) in the Arbequina olive pomace in the US. Moreover, 70% methanol is a common standard solvent for the analytical purpose of phenol extractions [20,21]. Water and 70% ethanol were also selected in this study and are considered as green, safe and nontoxic media for extraction compared to other organic solvents [22,23]. We are currently studying other olive cultivars in the US for a more comprehensive investigation, as Arbequina cultivars tend to have lower TPC compared to many other cultivars.

The yields of dried crude extracts from water, 70% methanol and 70% ethanol were 45.75%, 45.58% and 47.58% (g dry paste/g DOP), respectively. While almost half of the soluble matters were extracted, the TPC increased to 52.42 ± 2.21 mg GAE/g dry paste solids, 69.17 ± 2.10 mg/g and 65.83 ± 2.43 mg/g, by the three extraction solvents, respectively, which increased to about 1.5 times compared to the TPC in DOP (Figure 2).

To concentrate the TPC and eliminate free sugar impurities, Amberlite^®^ XAD7HP macroporous resin chromatography on pilot-scale was implemented for the purification. Because the XAD7HP has been reported to have 91% adsorption and 97% desorption of olive phenols [5], we selected it for the purification of the OP extract. TPC in the resin purified freeze-dried powders increased 4.6 times to 303.03 ± 6.74 mg GAE/g freeze-dried solids from 43.25 ± 2.08 mg/g of the 70% ethanol extracted dry paste (Figure 2). Although several studies [5,24,25] have reported the kinetics of macroporous resin absorbing OP phenols based on an analytical scale of several grams of resin, limited studies obtained freeze-dried powders of resin purified extract and reported the TPC. This study used preparative and pilot-scale resin column by filling hundreds of grams of resin, demonstrating that it may be feasible to scale-up the purification process to an industrial level.

### 3.3. Individual Phenolic Contents in Olive Pomace and Extracts

**Figure 3:**
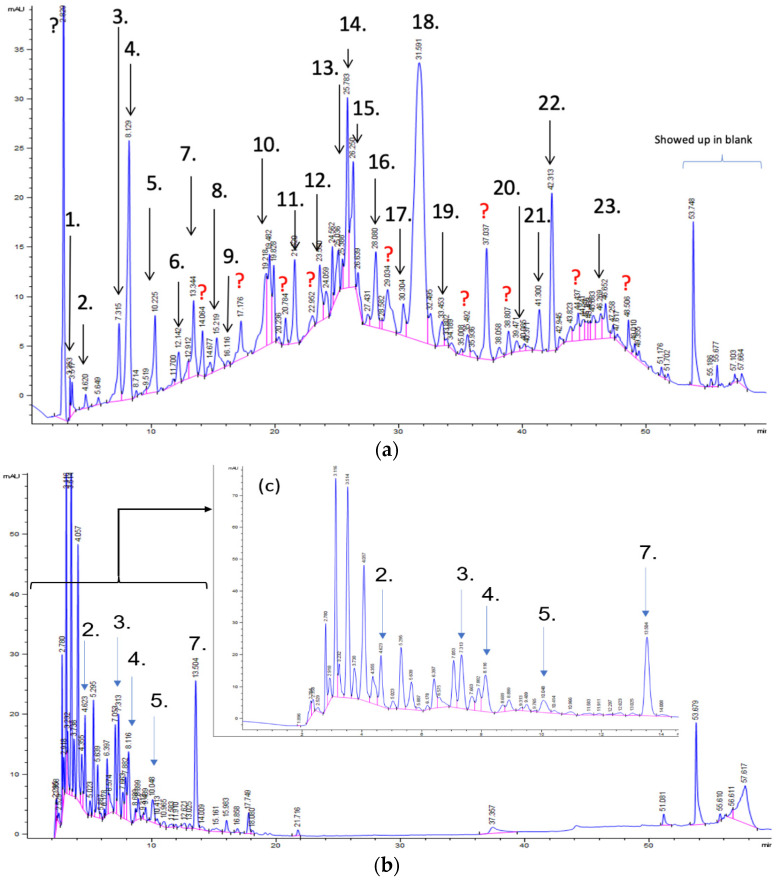
(**a**) Chromatography of XAD7HP resin purified freeze-dried powder at 280 nm and tentatively identified compounds by HPLC–DAD and HPLC-ESI-QToF-MS^n^; (**b**) Chromatography of the syrup of resin elutes during absorption; (**c**) 0–14 min of (**b**). Note: 1. Vanillin–glucoside-pentose-side, 2. Gallic acid, 3. Hydroxytyrosol-glucoside, 4. Hydroxytyrosol, 5. Tyrosol-glucoside, 6. Tyrosol, 7. 4-Hydroxyphenylacetate (4-HPA), 8. Vanillic acid, 9. Caffeic acid, 10. Vanillin, 11. P-coumaric acid, 12. Ferulic acid, 13. Verbascoside 14. Rutin, 15. Luteolin-7-glucoside, 16. O-coumaric acid, 17. Apigenin-7-glucoside, 18. 3,4-DHPEA-EDA, 19. Oleuropein, 20. Pinoresinol, 21. Cinnamic acid, 22. Luteolin, and 23. Apigenin. Mass spectrometric data refer to the Table 3. Spectra at 320, 365 nm and total ion chromatogram (TIC) refer Figure S1.

Figure 3a shows 19 phenolic compounds were identified by phenol standards, and these 19 phenolic compounds and 5 phenolic sugar derivatives and others were further putatively identified by mass spectrometry in Table 2. The tentatively identified phenols included unique compounds in olive and olive pomaces, such as hydroxytyrosol, tyrosol, 4-hydroxyphenyl acetic acid (4-HPA), verbascoside, oleuropein and their sugar derivatives, as well as several compounds commonly found in other plants, such as gallic acid, rutin and luteolin, etc. About ten unknown peaks have yet to be elucidated by HPLC-ESI-QToF-MS^n^.

**Figure 4:**
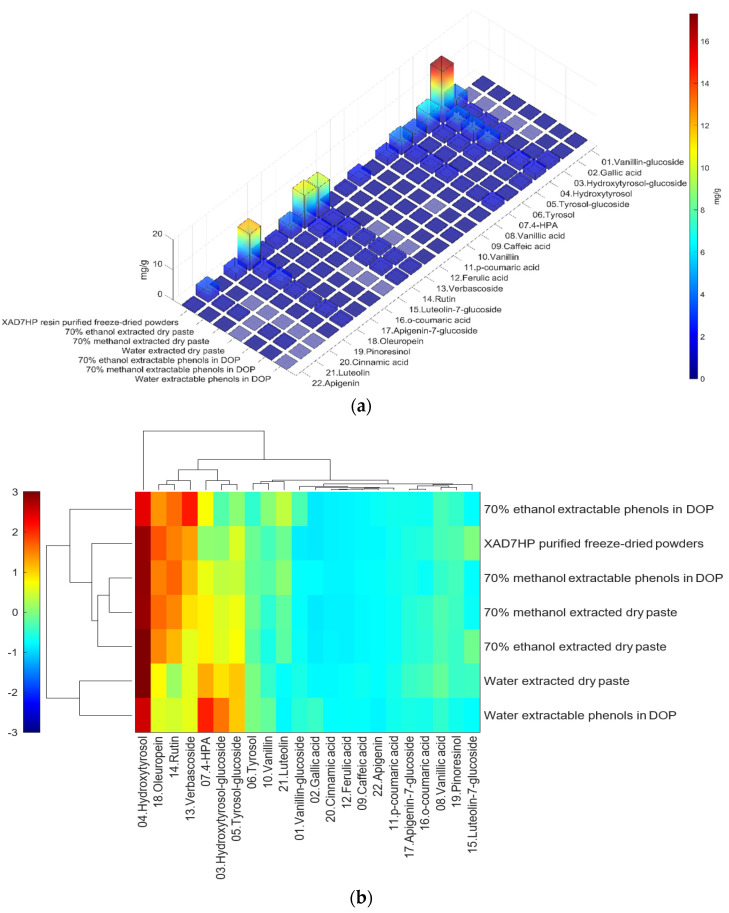
(**a**) Heatmap of individual phenolic contents (mg/g) of different extracted methods and purified steps; average and standard deviation can be found in Table 3; (**b**) heatmap cluster analysis of individual phenolic content of different extracted methods and purified steps; data were standardized along each row. Note: XAD7HP resin purified freeze-dried powder was made from 70% ethanol extracts, without oven-drying.

The concentration of individual phenolic compounds is in Table 3 and visualized in Figure 4a. The hydroxytyrosol, 4-HPA, rutin and many others of three extracted dry paste were about 2 times the extractable individual phenolics in DOP. In addition, those major individual phenolic compounds of the resin purified freeze-dried powders increased about 4–5 times as that of the 70% ethanol extracted dry paste. The results of individual phenolic compounds were in line with TPC discussed in the previous section. Although resin purification captured most of the OP phenolic compounds as shown in Figure 3a, some polar molecules in Figure 3 b and c (0–14 min) escaped from the resin column, which explained the TPC detected in syrup of resin elutes during absorption (Figure 2).

Figure 4b shows the heatmap cluster analysis where we transformed absolute values of individual phenolic contents to standardized values in each row for comparing phenolic compound profiles. By observing the ‘red hot zone’ of the heatmap, major compounds included hydroxytyrosol, oleuropein, rutin, verbascoside, 4-HPA, hydroxytyrosol-glucoside and tyrosol-glucoside. 3,4-DHPEA-EDA at the peak 18 in Figure 3a was found to possibly be the dominantly abundant phenol in the OP if the concentration was calculated to oleuropein equivalents. However, due to the lack of a standard of 3,4-DHPEA-EDA, the large area of the peak 18 could be attributed to the intensive response of the chemical to the UV at 280 nm, instead of an actual high concentration. Although 3,4-DHPEA-DEDA has been reported to be the most abundant phenol in a Spanish OP [26], we lack the confirmation of standard chemical and the standard curve to report the compound as the abundant phenol in the US OP.

The first tier of the vertical cluster generally classified samples to the water extracts group and alcoholic extracts group, respectively. Meanwhile, the orange-to-red ‘hot zone’ of the third–fourth tiers of the horizontal clusters showed the major water-extracted compounds were 4-HPA, hydroxytyrosol and tyrosol-glucoside which were relatively higher polar and water-soluble molecules, whereas the primary alcohol-extracted compounds were oleuropein, verbascoside and rutin which were relatively less polar and less water soluble. Furthermore, methanol and ethanol extracts were grouped to their subvertical clusters; however, the grouping trend was not entirely true for the ethanol-extracted dry paste because of phenolic compound profile difference. Compared with the fresh 70% extractable ethanol in DOP, the verbascoside content in ethanol-extracted dry paste decreased while the hydroxytyrosol increased, which indicated that the verbascoside decomposed and released its hydroxytyrosol sidechain during the oven drying. Similarly indicated compound shifts were also observed by the changes from 4-HPA and hydroxytyrosol-glucoside to hydroxytyrosol in water-extracted dry paste and from verbascoside to hydroxytyrosol in methanol extracted dry paste, respectively. The precursors of hydroxytyrosol and tyrosol, such as oleuropein tend to decompose into hydroxytyrosol under high temperatures [27]. Previously, our group [8] reported that hydroxytyrosol, tyrosol, caffeic acid, verbascoside and luteolin-7-glucoside were well preserved by drum-drying at 135 °C while oleuropein decomposed, which was in agreement with this study. Individual phenols in olive pomace from the production stream of virgin olive oil (VOO) in Span have been extracted by superheated liquid extraction at 160 °C for presenting 5 min [15], and they found many aglycon derivatives, such as *p*-HPEA-EDA derived from ligstroside and 3,4-DHPEA-EA derived from oleuropein, as well as decarboxymethylated aglycone derivatives, such as 3,4-DHPEA-EDA derived from oleuropein and p-HPEAEDA derived from ligstroside. However, not all those derivatives were found in this study, which may be due to the drum-drying process that decomposed those derivatives to more stable compounds such as hydroxytyrosol and tyrosol as well as their glucoside derivatives. In addition, although major olive phenols have been identified in this study, further study is still necessary to identify those unknown peaks in Figure 3a.

### 3.4. Removal of Sugar Impurity

Figure 2 shows sugar impurity accounted for 538.37 ± 33.39 mg glucose equivalents (GE)/g dry paste, 546.95 ± 22.73 mg/g and 598.69 ± 71.34 mg/g in water, 70% methanol, 70% ethanol extracts, respectively, which were the major components of the three dry pastes of the crude extracts. Although resin purified freeze-dried powders increased 4.6 times from that of the 70% ethanol-extracted dry paste, the total sugar content decreased by 37.33%. However, the total sugar content was still up to 375.20 ± 19.00 mg GE/g which may be primarily attributed to those phenol sugar derivates in Figure 3 instead of free sugars, because free sugars in water cannot be adsorbed by macroporous resin [28], theoretically, and should be removed during the DI water rinsing step [29]. While many previous studies [5,24,30] evaluated the purification of olive phenols by resin, few studies have revealed the removal of sugar impurity alone with the increment of olive phenols. This study provided a more comprehensive evaluation of the resin purification step, but further study on the individual free sugar molecules should be conducted to reveal the mechanism of the separations of sugars from phenols in olive pomace extracts.

### 3.5. In Vitro Antioxidant Activities of Olive Pomace Extracts

**Figure 5:**
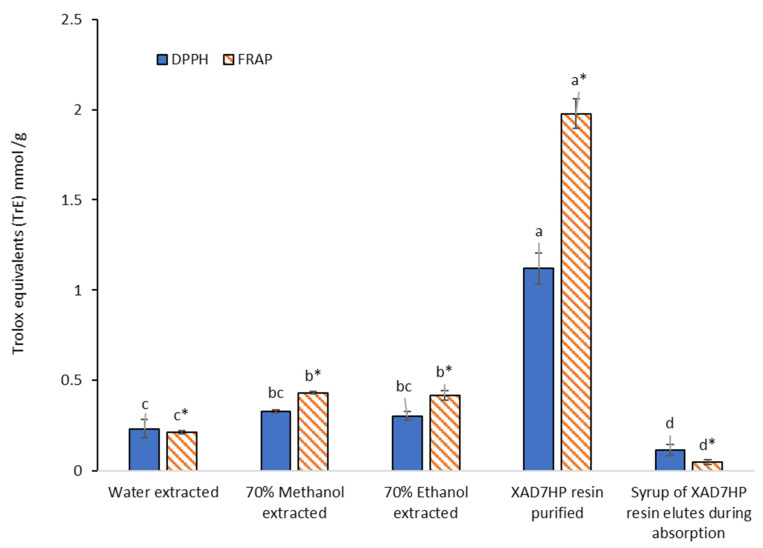
DPPH scavenging and FRAP activity among crude extracts, resin-purified extracts, different letters indicate significantly different, *p* < 0.05. Note: significant differences were only compared in either DPPH without”*” on significant markers or FRAP with “*” on significant markers. Data of syrup of the resin elutes were reported to dry matters (DM).

It can be seen from Figure 5, while the three crude extracts performed less than 0.5 TrE of both 2,2-diphenyl-1-picrylhydrazyl (DPPH) and ferric reducing antioxidant power (FRAP), XAD7HP resin purified freeze-dried powders increased 3.7 times TrE for DPPH and 4.7 times TrE for FRAP compared to that of the 70% ethanol extracted dry pasted. The results generally correlated with the 4.6 times increment in TPC, although the increments of DPPH were not proportionally correlated with the TPC increase. It has been reported that DPPH was not always consistent with other antioxidant activities [31] or proportional to TPC, because some phenolic compounds may be inactive to participate in scavenging DPPH radicals [32]. Very recently, three selected food models (flour, whole-wheat flour and sugar) were fortified by hydroxytyrosol from olive oil, while the oil and fat were removed by supercritical fluid carbon dioxide, leaving olive phenols in the foodstuffs and increasing the antioxidant activities of the food models [33]. This study purified phenols from olive pomace (OP) instead of olive oil, which would be a more cost-effective way for nutrition fortification by directly adding and distributing OP phenols into foodstuffs and their premix.

## 4. Conclusions

Our study demonstrated that major phenols in the Aberquina olive pomace from California are hydroxytyrosol, oleuropein, rutin, verbascoside, 4-HPA, hydroxytyrosol-glucoside and tyrosol-glucoside. Macroporous resin purification increased the total phenolic content (TPC) by 4.6 times the ethanol crude extracts of DOP while removing 37.33% of total sugar, while the extractable TPC was 36–43 mg GAE/g DOP. Meanwhile, the antioxidant activities increased 3.7 times TrE for DPPH and 4.7 times TrE for FRAP compared to the ethanol crude extracts. This new data on the chemical compositions of the selected US OP provides important and practical knowledge for the valorization and industrial food applications of the US olive wastes. Extracting bioactive compounds from the US OP not only could directly produce value-added products from food byproducts and increase consumers’ health via the delivery of natural antioxidants but also reduce negative environmental impact. The chemical and phenolic composition of more olive cultivars in the US should be determined and compared for better assessment of the valorization of the US OP.

## Figures and Tables

**Table 1:** Basic chemical composition analysis of Arbequina olive pomace.

Nutritional Component	Contents %
Protein	10.28 ± 0.11
Moisture	2.83 ± 0.08
Fat	11.72 ± 0.07
Ash	9.02 ± 0.04
Total carbohydrates	66.15

**Table 2:** Retention times and mass spectrometric data of tentatively identified compounds by HPLC-ESI-QToF-MS^n^.

No.	Tentatively Identified Compounds	Retention Time (min)	MW/Da	MS (*m*/*z*) [M-H]^−^	MS/MS (*m*/*z*)	Molecular Structure
01	Vanillin-glucoside	3.25	448.15	447.15	447.15, 315.11, 169.05, 153.06, 123.05	NA
02	Gallic acid	4.62	170.05	169.05	141.02, 123.01	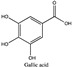
03	Hydroxytyrosol-glucoside	7.32	316.11	315.11	153.06, 123.05, 101.02	NA
04	Hydroxytyrosol	8.13	154.16	153.06	123.05, 109.03, 101.02	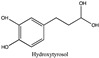
05	Tyrosol-glucoside	10.23	300.14	299.14	183.06, 139.07, 119.04, 101.02	NA
06	Tyrosol	12.14	138.16	137.16	119.04, 101.03	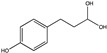
07	4-Hydroxyphenylacetate (4-HPA)	13.34	152.15	151.04	163.04, 123.05	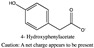
08	Vanillic acid	15.22	168.03	167.03	ND	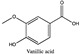
09	Caffeic acid	16.12	180.06	179.06	ND	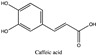
10	Vanillin	19.22	152.15	151.04	123.05, 139.00	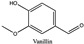
11	p-coumaric acid	21.62	164.04	163.04	119.04, 101.02	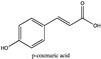
12	Ferulic acid	23.38	195.06	194.06	151.04, 135.04	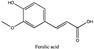
13	Verbascoside	25.78	624.59	623.20	458.20, 461.15, 151.04	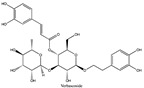
14	Rutin	25.25	610.52	609.52	609.15, 301.03	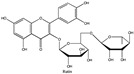
15	Luteolin-7-glucoside	26.25	448.09	447.09	285.04	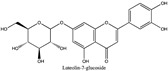
16	o-coumaric acid	28.08	165.05	164.05	ND	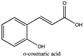
17	Apigenin-7-glucoside	30.30	432.38	431.38	ND	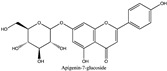
18	3,4-DHPEA-EDA	31.59	320.12	319.12	139.08, 123.05	NA
19	Oleuropein	33.45	540.18	539.18	377.12, 307.08, 275.09	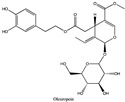
20	Pinoresinol	39.47	358.10	357.10	341.12, 151.04	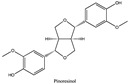
21	Cinnamic acid	41.30	148.16	147.16	ND	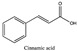
22	Luteolin	42.31	286.04	285.04	217.00	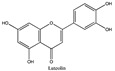
23	Apigenin	46.27	270.04	269.04	241.07, 141.02	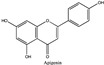

Note: ND means ms/ms was not detected by HPLC-QToF-MS^n^, but the compound was identified by HPLC-DAD with its standard chemical by retention time. NA means not available. Retention time were matched to HPLC-DAD in Figure 3a.

**Table 3:** Individual phenolic contents (mg/g of sample) of different extracted methods and purified steps of olive pomace.

Name	Water Extractable Phenols in DOP	70% Methanol Extractable Phenols in DOP	70% Ethanol Extractable Phenols in DOP	Water Extracted Dry Paste	70% Methanol Extracted Dry Paste	70% Ethanol Extracted Dry Paste	XAD7HP Resin Purified Freeze-Dried Powders
01. Vanillin-glucoside	0.165 ± 0.004	0.054 ± 0.002	0.225 ± 0.021	0.151 ± 0.001	0.155 ± 0.001	0.152 ± 0.000	0.187 ± 0.002
02. Gallic acid	0.223 ± 0.006	0.045 ± 0.004	0.000 ± 0.009	0.007 ± 0.002	0.008 ± 0.011	0.010 ± 0.003	−0.018 ± 0.026
03. Hydroxytyrosol-glucoside	1.407 ± 0.034	0.657 ± 0.024	0.250 ± 0.006	1.423 ± 0.006	1.475 ± 0.034	1.480 ± 0.003	4.423 ± 0.278
04. Hydroxytyrosol	1.978 ± 0.039	2.017 ± 0.089	1.356 ± 0.054	3.508 ± 0.500	3.880 ± 0.027	4.219 ± 0.459	17.298 ± 0.363
05. Tyrosol-glucoside	1.096 ± 0.022	0.679 ± 0.026	0.384 ± 0.018	1.555 ± 0.031	1.581 ± 0.006	1.639 ± 0.085	6.519 ± 0.421
06. Tyrosol	0.460 ± 0.008	0.365 ± 0.008	0.162 ± 0.012	0.624 ± 0.014	0.811 ± 0.091	0.666 ± 0.002	3.514 ± 0.060
07. 4-HPA	1.700 ± 0.044	0.800 ± 0.027	0.660 ± 0.023	1.691 ± 0.005	1.755 ± 0.013	1.742 ± 0.010	4.450 ± 0.021
08. Vanillic acid	0.203 ± 0.004	0.208 ± 0.007	0.223 ± 0.004	0.509 ± 0.044	0.609 ± 0.009	0.585 ± 0.001	2.530 ± 0.018
09. Caffeic acid	0.050 ± 0.001	0.044 ± 0.002	0.039 ± 0.002	0.073 ± 0.011	0.102 ± 0.009	0.091 ± 0.001	0.420 ± 0.001
10. Vanillin	0.371 ± 0.005	0.329 ± 0.014	0.375 ± 0.011	0.285 ± 0.044	0.385 ± 0.006	0.269 ± 0.001	2.439 ± 0.027
11. p-coumaric acid	0.084 ± 0.006	0.097 ± 0.003	0.086 ± 0.004	0.131 ± 0.001	0.168 ± 0.001	0.157 ± 0.000	0.884 ± 0.002
12. Ferulic acid	0.047 ± 0.001	0.023 ± 0.002	0.029 ± 0.001	0.043 ± 0.003	0.046 ± 0.000	0.047 ± 0.000	0.326 ± 0.012
13. Verbascoside	0.833 ± 0.007	1.074 ± 0.035	1.232 ± 0.037	1.135 ± 0.003	1.858 ± 0.016	1.507 ± 0.000	10.159 ± 0.052
14. Rutin	0.770 ± 0.011	1.360 ± 0.050	1.031 ± 0.035	0.791 ± 0.019	2.409 ± 0.216	2.108 ± 0.120	11.048 ± 0.003
15. Luteolin-7-glucoside	0.042 ± 0.000	0.042 ± 0.000	0.042 ± 0.000	0.312 ± 0.236	0.175 ± 0.000	0.785 ± 0.529	4.086 ± 0.022
16. o-coumaric acid	0.101 ± 0.000	0.070 ± 0.000	0.070 ± 0.000	0.352 ± 0.041	0.416 ± 0.004	0.369 ± 0.001	1.562 ± 0.005
17. Apigenin-7-glucoside	0.055 ± 0.000	0.121 ± 0.003	0.088 ± 0.001	0.293 ± 0.012	0.341 ± 0.002	0.336 ± 0.001	1.345 ± 0.034
18. Oleuropein	0.811 ± 0.012	1.270 ± 0.324	0.930 ± 0.093	1.298 ± 0.188	2.609 ± 0.073	2.393 ± 0.052	12.231 ± 0.066
19. Pinoresinol	0.084 ± 0.001	0.257 ± 0.005	0.175 ± 0.012	0.300 ± 0.087	0.478 ± 0.136	0.461 ± 0.025	2.775 ± 0.495
20. Cinnamic acid	0.027 ± 0.002	0.019 ± 0.000	0.013 ± 0.004	0.012 ± 0.000	0.043 ± 0.024	0.063 ± 0.004	0.205 ± 0.111
21. Luteolin	0.010 ± 0.000	0.487 ± 0.016	0.515 ± 0.034	0.041 ± 0.000	0.714 ± 0.003	0.678 ± 0.001	3.515 ± 0.003
22. Apigenin	0.007 ± 0.000	0.062 ± 0.004	0.066 ± 0.004	0.030 ± 0.000	0.111 ± 0.006	0.107 ± 0.007	0.469 ± 0.031

Note: Vanillin-glucoside, hydroxytyrosol-glucoside and tyrosol-glucoside are expressed by equivalents of vanillin, hydroxytyrosol and tyrosol, respectively.

## Data Availability

The data presented in this study are available in within the article and Supplementary Materials.

## Supplementary Materials

The following supporting information can be downloaded at: https://www.mdpi.com/article/10.3390/foods11020174/s1, Figure S1: Chromatography of XAD7HP resin purified freeze-dried powder (**a**) at 320 and (**b**) at 365 nm and (**c**) total ion chromatogram (TIC).
