# Supplementary material for: Extraction, Purification and In Vitro Antioxidant Activity Evaluation of Phenolic Compounds in California Olive Pomace
Source: Foods. 2022 Jan 10;11(2):174. doi: 10.3390/foods11020174 (PMC8775219; doi:10.3390/foods11020174)
Supplement: Supplementary file 1 [file foods-11-00174-s001.zip › foods-1518175-supplementary.pdf]

Supplementary

Extraction, Purification, and in vitro Antioxidant Activity Evaluation of Phenolic Compounds in California Olive Pomace

Hefei Zhao[1], Roberto J. Avena-Bustillos[2], Selina C. Wang[1,3] *

[1] Department of Food Science and Technology, University of California, Davis, CA 95616, USA
[2] Western Regional Research Center, Healthy Processed Foods Research, Albany, CA, USA
[3] Olive Center, University of California, Davis, CA 95616, USA
* Correspondence: scwang@ucdavis.edu

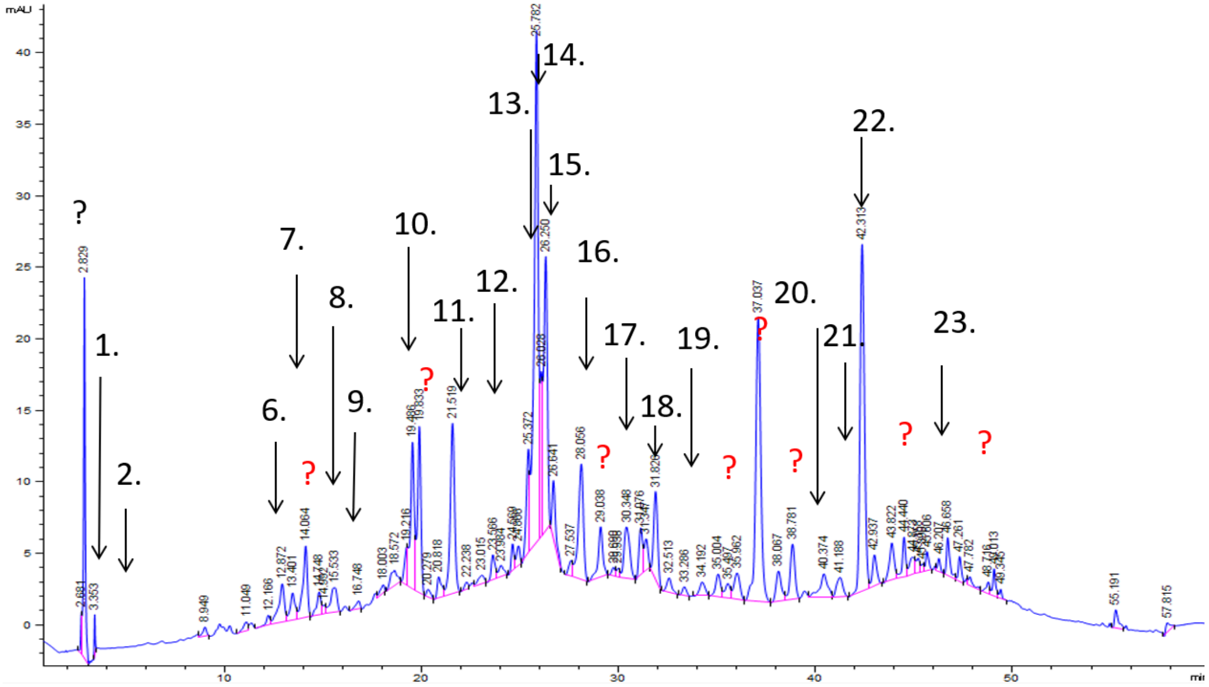

(a)

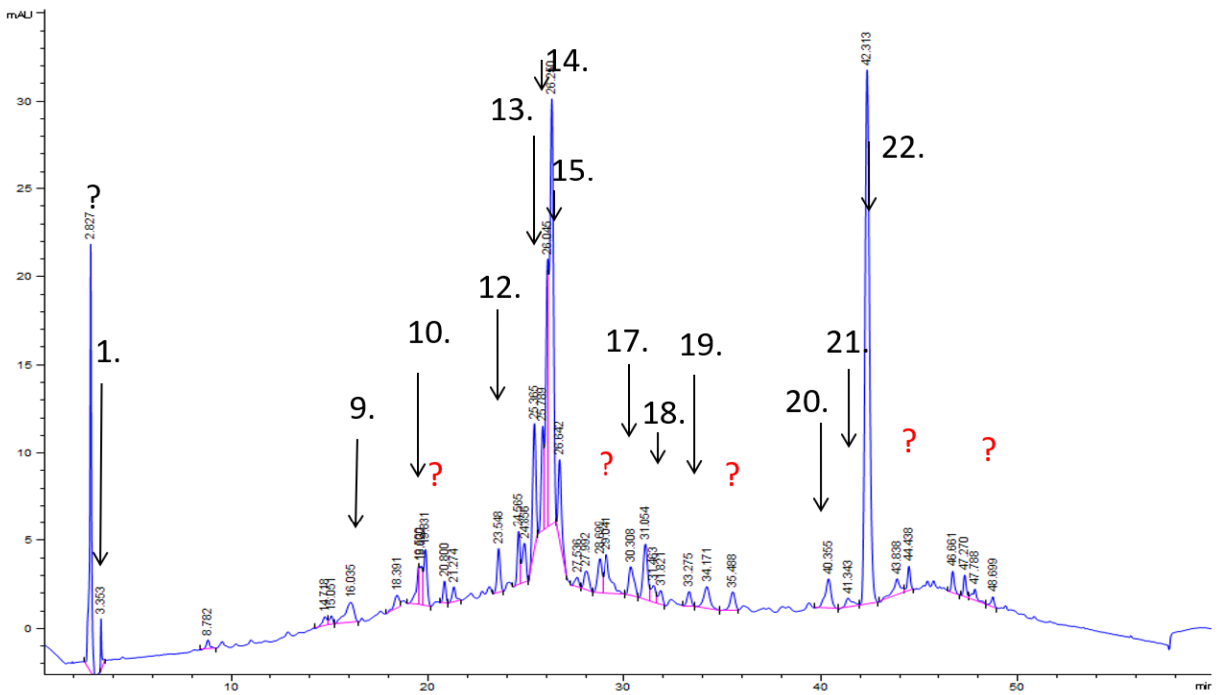

(b)

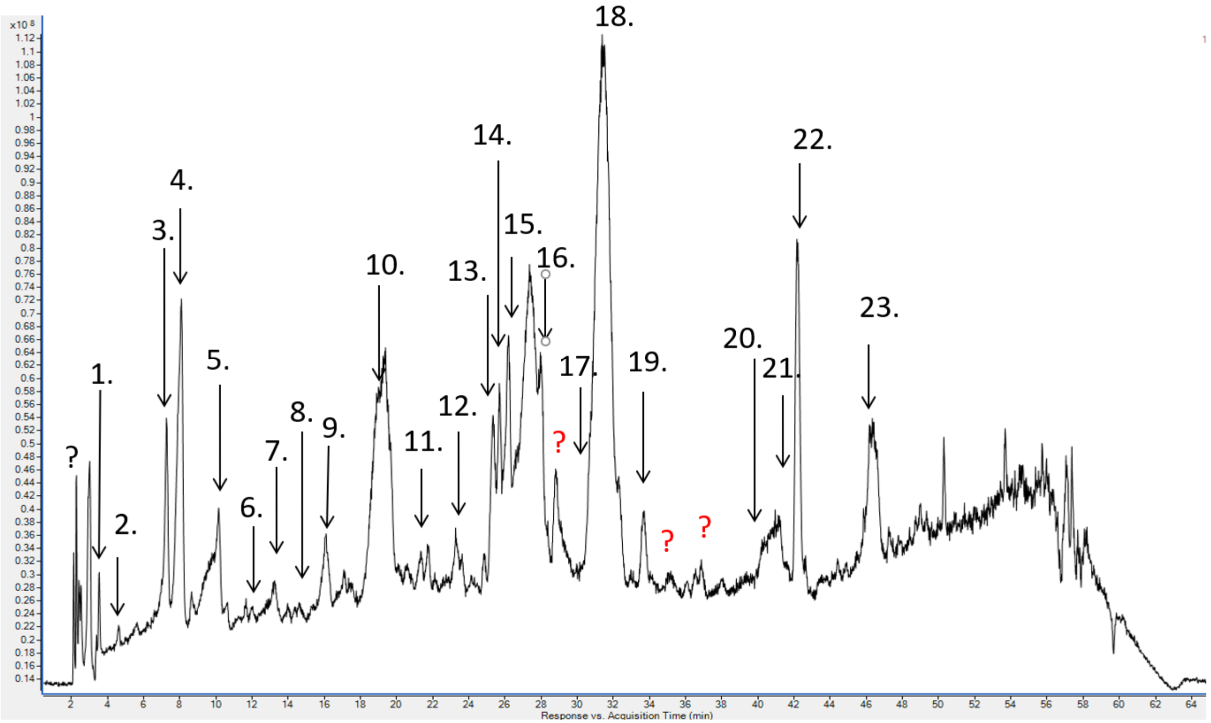

(C)

Figure S1. Chromatography of XAD7HP resin purified freeze-dried powder (a) at 320 and (b) at 365 nm and (c) total ion chromatogram (TIC).
